# Human Milk Microbiota and Oligosaccharides: A Glimpse into Benefits, Diversity, and Correlations

**DOI:** 10.3390/nu13041123

**Published:** 2021-03-29

**Authors:** Carole Ayoub Moubareck

**Affiliations:** College of Natural and Health Sciences, Zayed University, Dubai 19282, United Arab Emirates; Carole.AyoubMoubareck@zu.ac.ae; Tel.: +971-4402-1745

**Keywords:** human milk, breastfeeding, human milk benefits, milk microbiota, human milk oligosaccharides, maternal diet

## Abstract

Human milk represents a cornerstone for growth and development of infants, with extensive array of benefits. In addition to exceptionally nutritive and bioactive components, human milk encompasses a complex community of signature bacteria that helps establish infant gut microbiota, contributes to maturation of infant immune system, and competitively interferes with pathogens. Among bioactive constituents of milk, human milk oligosaccharides (HMOs) are particularly significant. These are non-digestible carbohydrates forming the third largest solid component in human milk. Valuable effects of HMOs include shaping intestinal microbiota, imparting antimicrobial effects, developing intestinal barrier, and modulating immune response. Moreover, recent investigations suggest correlations between HMOs and milk microbiota, with complex links possibly existing with environmental factors, genetics, geographical location, and other factors. In this review, and from a physiological and health implications perspective, milk benefits for newborns and mothers are highlighted. From a microbiological perspective, a focused insight into milk microbiota, including origins, diversity, benefits, and effect of maternal diet is presented. From a metabolic perspective, biochemical, physiological, and genetic significance of HMOs, and their probable relations to milk microbiota, are addressed. Ongoing research into mechanistic processes through which the rich biological assets of milk promote development, shaping of microbiota, and immunity is tackled.

## 1. Introduction to Human Milk and Importance of Breastfeeding

Over the past decades, human breast milk has been widely agreed upon as being the normal and optimal dietary start for infants, with unparalleled biological effects driven by the combined action of its nutritional and bioactive components [1]. The positive effects of breastfeeding on infants from the nutritional, physiological, and developmental viewpoints have been confirmed [2]. Worldwide, health authorities have recognized the value and benefits of breastfeeding [3]. The World Health Organization (WHO) and the United Nations Children’s Fund (UNICEF) recommend the initiation of breastfeeding within the first hour of birth and advise on exclusive breastfeeding for the first six months of life, without any other food or liquids, including water. Although the introduction of complementary food is safe starting at six months, the WHO and UNICEF recommend to pursue breastfeeding for up to two years [4]. In the breastfeeding week 2020 message, under the theme “support breastfeeding for a healthier planet”, the WHO called for protection, promotion, and support of women’s access to skilled breastfeeding, in line with the multifaceted benefits of this sustainable, biological food system [5]. Likewise, the American Academy of Pediatrics (AAP) continuously reinforces breastfeeding and mother’s milk as the normal standards for infant feeding and nutrition. It recommends exclusive breastfeeding for six months, followed by continuation of breastfeeding as complementary foods are introduced, with possible extension of breastfeeding for one year of age or beyond, as desired [6].

Human milk holds a plethora of health benefits, both on the short- and the long-term. The relationship between breastfeeding and infant’s health is based on its nutritional and non-nutritional components that have diverse roles [7]. Human milk contains the precise mix of nutrients, including carbohydrates, proteins, fats, vitamins, minerals as well as water, that together secure a dynamic composition, well developed to fulfil needs of the infant growth [8]. The main nutrient constituents of milk are shown in Table 1 [8,9,10,11]. Further to nutrients, human milk contains many bioactive constituents like immunoglobulins, growth factors, microRNAs, and human milk oligosaccharides (HMOs). These form a complex system linking the mother’s lifestyle, such as diet and gut microbiota, with outcomes on infant growth, gut microbiota, immunity, and other developmental features [12]. In the infantile gut, a human milk-directed microbiota develops, forming initial doses for seeding the mature gut microbiome. Additionally, the high concentration and structural diversity of HMOs that reach the infant colon, initiate a series of health effects [13]. To this end, this review article presents up-to-date information about human milk benefits, human milk microbiota, and HMOs, and revises possible associations between microbial milk profiles and both maternal diet and HMOs.

## 2. Human Milk Benefits

A priceless natural regimen presented by a mother to her newborn, human milk offers an extensive array of health benefits [14]. Milk from mothers whose diet is sufficient and well-balanced supplies all the necessary nutrients suitable for ideal growth (except vitamin D, whose intake should be 400 IU/day for all breastfed infants) [15]. Any amount of breastfeeding is better than none, and superior benefits accumulate with increased duration [3]. In addition to its supreme nutritive value, current scientific evidence about breastfeeding supports many beneficial effects, not only for infants who are breastfed on both short- and long-term, but also for mothers who breastfeed [16].

### 2.1. Short-Term Benefits for Infants

Breastfeeding possesses obvious short-term benefits for infant health, mostly in decreasing mortality and morbidity resulting from infectious diseases, such as gastrointestinal and respiratory tract infections, as well as otitis media, with a level of evidence described as convincing [15].

The protection conferred by breastfeeding against diarrhea is certainly one of the most consistent findings in the epidemiological literature [3,15,17,18,19]. A systematic review published by the WHO in 2013 suggests that breastfeeding substantially protects against morbidity/mortality from diarrhea, with a reduction of 50% in morbidity and 80–90% in mortality and hospital admissions, compared to infants with less or no breastfeeding. Such protection was higher among infants who were exclusively breastfed in the first six months of life and was provided by more intense breastfeeding. These robust results were observed across high and low-income settings [17]. An integrative review by Santos and Colleagues [20] in 2015 also replicated similar results, confirming the importance of breast feeding in the prevention of diarrhea in children under six months, especially among those who were exclusively breastfed. Such studies reinforce the recommendations for exclusive breastfeeding during the first six months of life as a key intervention for infant survival. The protective effect of breastfeeding against gastrointestinal infections has been attributed to the positive role of the microbial and immunological milk constituents and to the lack of contamination from baby bottles [19].

In addition to prevention of diarrhea, it is well documented that breastfeeding is associated with a significantly lower risk for lower respiratory tract infections and otitis media, compared with non-breastfed children [21]. Breast milk is associated with around 30% reduction in morbidity, 50% in hospital admissions, and 60% in mortality from these infections, suggesting that breastfeeding affects not only the incidence of these infections, but also their severity [17]. A systematic literature review and meta-analysis showed that suboptimal breastfeeding increased the risk of morbidity and mortality from pneumonia across different infant age groups. In particular, pneumonia mortality was higher among non-breastfed compared to exclusively breastfed infants 0–5 months of age, and among non-breastfed compared to breastfed infants and young children 6–23 months of age [22]. In a study from UK, higher risk of respiratory infection was found among infants exclusively breastfed for 4–6 months, but who stopped breastfeeding by six months, adding to the evidence of continued milk benefits after six months [23]. Furthermore, evidence shows that breastfeeding protects against acute otitis media until two years of age, but protection is greater for exclusive breastfeeding and breastfeeding of longer duration [24]. In a recent cohort analysis from Denmark in 2020, involving 815 mother-infant pairs, a strong association between breastfeeding and hospitalizations due to lower respiratory tract infection was found particularly in the first year of life [25]. In an interventional study to promote successful breastfeeding, Zivich and Colleagues showed that breastfeeding reduced incidence of mild as well as severe episodes of both respiratory infection and diarrhea in the first six months of life [26].

Recently, with the upsurge of Coronavirus Disease 2019 (COVID-19), caused by the severe acute respiratory syndrome coronavirus 2 (SARS-CoV-2), recommendations about breastfeeding in infected mothers were endorsed [27]. Breastfeeding in postpartum women with SARS-CoV-2 is recommended by the WHO [28] and by the US Centers for Disease Control and Prevention (CDC) [29] for the newborn as long as health of the mother and newborn allow it. This belongs to the same context of the protective effect of mother’s milk against infections and is built on the general evidence that benefits of breastfeeding substantially outweigh the potential risks for SARS-CoV-2 transmission. Appropriate respiratory hygiene measures for infected mothers always have to be considered with direct breastfeeding [30].

Apart from infection, the American Academy of Family Physicians reports reduced incidence of atopic dermatitis (eczema) in breastfed infants [3]. Although there is evidence that exclusive breastfeeding for 3 to 4 months reduces incidence of atopic dermatitis in the first two years of life, long-term advantages for exclusive breastfeeding for prevention of atopic skin disease are not clear. Furthermore, there are no definite conclusions regarding the role of breastfeeding in preventing nor postponing the onset of specific types of food allergies in newborns [31]. In a nationwide cohort studied in Japan in 2020, breastfeeding had prophylactic effects on food allergy only among high-risk children (defined as having positive family history and eczema presentation prior to one year) with infantile eczema, whereas prolonged breastfeeding may increase the risk of food allergy [32].

Another possible benefit of human milk is being a protective factor against sudden infant death syndrome, which is the leading death cause in the United States in infants of ages 1 month to 1 year [33]. Several literature reviews have documented that sudden infant death syndrome risk decreases with breastfeeding [34,35,36], and the effect is stronger when breastfeeding is prolonged [37]. Such positive effect of breastfeeding has been deliberated in physiologic sleep studies, which have revealed that breastfed infants have lower arousal thresholds than their formula-fed counterparts, probably providing a survival mechanism for protection against this condition [38,39].

### 2.2. Long-Term Benefits for Infants

In addition to growth and infection prevention on the short-term, research into human milk also indicates that it may influence noncommunicable diseases or conditions later in life, perhaps offering a lifetime of benefits [40]. For instance, the relationship between breastfeeding and obesity prevention has long been sought by researchers. According to meta-analysis, breastfeeding appears to a significantly protect against obesity in childhood, although evidence on this role is still controversial, and the underlying mechanisms are still unclear [41,42]. Recently, in a prospective six-year study from Spain that followed a cohort of infants from birth up to six years of age, full breastfeeding was associated with a significant decrease in obesity. The delay in introduction of bottle feeding showed a probable protective effect against obesity at 6 years of age [43]. In trying to explain the role of breastfeeding in prevention of obesity, epidemiological data illustrate that breastfed children have healthier dietary patterns compared to formula-fed children, probably reducing their risk of obesity. These healthier patterns are, in part, caused by early opportunities for flavor learning afforded by breastfeeding. In particular, the flavors of the mother’s diet are transmitted to the infant via breastmilk, providing an additional reinforcement and flavor learning upon repeated exposure to a wide variety of flavors. The flavors experienced by the infant modulate later food preferences, acceptance of solid food, and culture onto which the infant is weaned [44]. Plus, breastfeeding favors the establishment of diverse microbial communities in infant’s gut that play a crucial role in preventing obesity via their various mechanisms of harvesting energy from food, affecting patterns of fatty acid oxidation, modulating bile acid metabolism, and influencing satiety and lipogenesis. It is still debatable to define which microbial category is responsible for obesity, and studies in this regard show discrepancies [45].

Studies linking human milk to protection against gastrointestinal tract diseases are also numerous. For the past two decades, human milk have been associated with reduced incidence in necrotizing enterocolitis in preterm infants [46,47]. Such infants’ susceptibility to enterocolitis stems from their gastrointestinal and immune system immaturity. It is thought that an exclusive human milk diet, consisting of mother’s own milk and/or donor human milk alone or fortified with a human milk-based fortifier, compensates for these immature systems by many pathways like stimulating intestinal motility, composed from lowering gastric pH, decreasing epithelial penetrability, and altering the composition of the gut microbiota [48]. In a multicenter study in a cohort of over 1000 patients with Crohn’s disease, a history of breastfeeding was inversely associated with complicated pediatric symptoms [49]. The evidence of the link between Crohn’s disease prevention and breastfeeding, however, is described as insufficient, and more reliable data are needed [15]. Similarly, limited evidence exists for the role of breastfeeding in reducing incidence of celiac disease, and further investigation is needed to indicate whether such protection only delays the onset of the disease or if it can provide permanent protection [50].

Metabolic disorders and tumors also had their share in research trying to investigate late benefits of breastfeeding. In a meta-analysis, Horta and Colleagues [40] found that breastfeeding decreased the odds of type 2 diabetes mellitus and based on high-quality studies, but no associations were found for total cholesterol or blood pressure. Breast milk has long-chain polyunsaturated fatty acids (LCPUFAs), whose supplementation is associated with a reduction in blood pressure. In addition, LCPUFAs would induce early changes in skeletal muscle which can be protective against insulin resistance and type 2 diabetes [51]. As far as cancer is concerned, and with leukemia, which accounts for 30% of childhood cancers, a meta-analysis showed that breastfeeding for six months or longer may help reduce the incidence of childhood leukemia by up to 19% [52]. Furthermore, a pediatric oncology team assessed relation between breastfeeding and cancer in a group of 300 children with cancer (leukemia, lymphoma, and solid organ cancers including brain, bone, liver, kidney, and others), and an approximately equal number of age- and sex-matched controls. Breastfeeding duration of the control group was found to be significantly longer than the patient group [53].

Perhaps the possible influence of breastfeeding on cognitive development is a subject that has not ceased to provoke considerable scientific debate. Despite substantial disparity of reported results, a positive correlation between breastfeeding and the levels of intelligence and cognitive development in children exists, although with a number of confounders like maternal age, education, socioeconomic status, and IQ [15,21]. In general, children who are breastfed for longer than six months are known to show better cognitive outcomes and a lower risk to develop attention-deficit/hyperactivity disorder [54]. Researchers from McGill University, and based on the largest randomized, interventional trial ever conducted in the field of human milk benefits, provide robust evidence that prolonged, exclusive breastfeeding enhances children’s cognitive development up to age of 6.5 years [55]. However, in a study from UK analyzing children till age of nine years breastfeeding was not associated with IQ advantage nor difference in neurological soft signs. The higher IQ associated with breastfeeding was probably accounted for by some confounding maternal and socio-economic factors [56]. The actual mechanisms linking improved cognitive development with breastfeeding remain unknown; however, it is suggested that human milk LCPUFAs are incorporated in relatively large amounts during early growth of the brain and the retina [57], and more specifically, the white matter of the brain [21]. Some of these acids, such docosahexaenoic acid (DHA) and arachidonic acid (ARA), present in human milk, may play a role in growth and development of eye, brain and nerve; these acids also exhibit potent biological activity that modulates various cellular and tissue processes [58,59]. Interestingly, and also in the field of neurologic development, a recent meta-analysis in 2020 concluded that breastfeeding for 12–24 months was associated with a significant decrease in autism spectrum disorder risk [60]. Furthermore, studies show that breastfed infants, as compared to bottle-fed infants, show higher vigor, including social approach, activity and the intensity of reaction [61]. The duration of breastfeeding was shown to correlate negatively with parent-reported antisocial conduct and aggressive behavior in children aged 4 to 11 years [62]. These and other findings continue to verify that human milk is not simply a meal at the breast, but rather a biological fluid with significant and far-reaching effects into child’s health, psychology, and social behavior.

It is worth noting that the public health significance of breastfeeding originates from a big pool of studies that are mostly observational. Large interventional or randomized controlled trials on breastfeeding are usually hindered by ethical issues. Therefore, the evidence for short-term outcomes mentioned above, such as reduced rates of infection, can be classified as strong. However, the longer term effects are probably less firm, due to difficulty in completely accounting for confounding factors [16].

### 2.3. Maternal Benefits

While the nutritional and physical health benefits conveyed by human milk are well established for infants, accumulating research reveals that breastfeeding mothers benefit as well. As an evidence of such profits, it was reported that mothers with premature weaning have health risks of breast and ovarian cancers, hypertension, hyperlipidemia, diabetes mellitus, myocardial infarction, and obesity, with these risks being highest in those who do not breastfeed at all [63].

Breastfeeding reduces overall risk of breast cancer [64]. In a meta-analysis, exclusive breastfeeding among parous women was found to reduce the risk of breast cancer compared with parous women who do not exclusively breastfeed [65]. Furthermore, mothers positive for the BRCA1 mutation and breastfeeding for at least one year have 37% lower breast cancer risk compared to carriers of the mutation who do not breastfeed [66]. The reduced risk of breast cancer is most evident in postmenopausal women and is directly proportional to the duration of lactation. The protective effect of breastfeeding is attributed to differentiation of breast cells, decreased number of ovulatory cycles, and estrogen excretion through milk, reducing overall exposure to this hormone [67]. In addition to breast cancer, evidence suggests that breastfeeding reduces risk of ovarian cancer [63]. Modugno and Colleagues [68] concluded that breastfeeding for as few as three months was associated with reduced risk of ovarian cancer, and the association was long-lived. The probable mechanism of breastfeeding in ovarian cancer protection may be due to absence of ovulation. Monthly ovulation might increase the odds of genetic mutations, and ovarian overstimulation by elevated gonadotropins may trigger hyperproliferation, with possible malignant transformation [69].

Prolonged breastfeeding has beneficial effects in reducing the long-term risk of coronary artery disease. Parous women who breastfeed for five or more months in at least one pregnancy seem to be at decreased risk of coronary artery disease, whereas parous women who either did not breastfeed at all or discontinued breastfeeding early seem to be at elevated risk [70]. The European Society of Cardiology concluded that breastfeeding women had a lower risk of coronary artery disease later in life compared to those who do not breastfeed [71]. Data from studies in both animals and humans suggest that lactation may alter maternal sugar and lipid homeostasis and may exert effects on blood pressure regulation. This might have significant effects on lipid homeostasis, with lower triglyceride and higher HDL cholesterol levels, possibly reducing risks of heart diseases [72]. Breastfeeding also affects maternal risks of hypertension and diabetes mellitus [63,64,73]. Park and Choi [74] studied a population of over 3000 nonsmoking postmenopausal women aged 50 years or above, and showed that more children breastfed and longer breastfeeding duration were associated with lower risk of hypertension. A systematic review and meta-analysis determined that variable breastfeeding durations have different protective effects against maternal hypertension, especially if continued beyond 12 months [75]. Longer duration of breastfeeding, whether full or partial, is associated with lower maternal risk of hypertension and heart disease irrespective of pre-pregnancy body mass index and abdominal adiposity seven years after delivery [76]. Further, mothers who breastfeed have less visceral obesity and smaller waist circumference, probably accounting for a lower maternal risk of type 2 diabetes mellitus [77], and this was recently proved in a prospective cohort of women followed for 14.2 years [78]. Few common mechanisms of relationships between breastfeeding and maternal risks of hypertension and diabetes were suggested. For example, maternal metabolism (e.g., fat accumulation and insulin resistance) may be reversed by breastfeeding, which decreases both diseases risk. During pregnancy, visceral fat accumulates, insulin resistance rises, and lipid and triglyceride levels increase. These changes appear to retune more rapidly, and more entirely, with lactation—the so called “reset hypothesis” [79]. Another theory is that oxytocin, needed in glucose homeostasis and the release of which is stimulated by breastfeeding, may be associated with decreased risk of type 2 diabetes mellitus [80]. It is worth mentioning that milk production, which consumes about 500 kcal per day for an infant who is exclusively breastfed, reduces maternal obesity later in life [81].

Apart from cardiovascular health, breastfeeding affects psychological well-being. Breastfeeding mothers report less anxiety, less negative mood, and less stress, as well as increased sleep duration and reduced sleep disturbances when compared to formula-feeding mothers [82,83]. On another note, studies on post-partum depression demonstrate that breastfeeding may protect mothers from this disorder, and researchers have strived to explain the biological processes that explain this protection. For example, lactation attenuates neuro-endocrine responses to stress, and this may be related to fewer post-partum depressive symptoms [84,85]. Moreover, early breastfeeding cessation was linked to higher risk of post-partum depression [86]. It is the psychological pressure to exclusively breastfeed that contributes to postpartum depression symptoms in mothers unable to achieve their breastfeeding intentions [87,88]. In a prospective follow-up for eight weeks postpartum, mothers with breastfeeding problems (including mastitis, nipple pain, need for frequent expressing of milk, or over-supply or under-supply of milk) showed poor mental health [89].

With all the aforementioned benefits of human milk on infant and maternal wellness, breastfeeding continues to form a cornerstone for prevention of many short- and long-term health risks. It is a real investment in health, rather than a mere lifestyle decision. The promotion of human milk as a highly active biological milieu is substantially imperative, and further research into its numerous potential benefits is always appealing.

**Figure 1:**
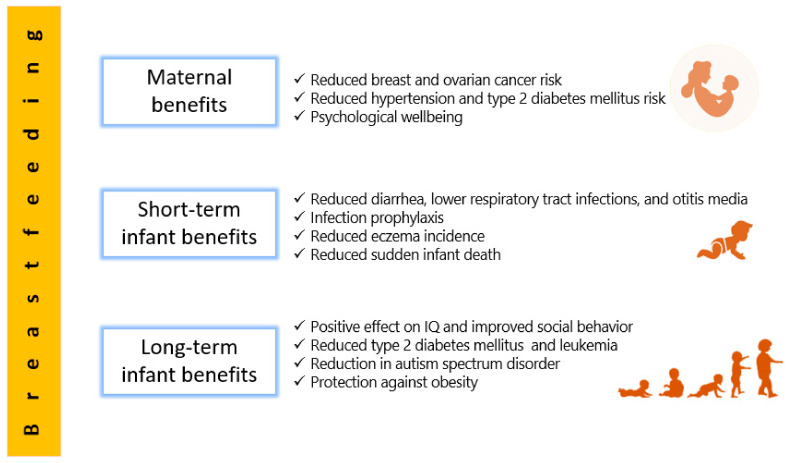
A summarized description of the benefits of breastfeeding and effects of human milk on the early and long-term infant health, as well as benefits for the mother.

Figure 1 summarizes data from Section 2.1, Section 2.2 and Section 2.3 of this review pertaining to benefits of human milk and breastfeeding.

## 3. A Focused Insight into Human Milk Microbiota

In addition to its diverse nutritional and health benefits, human milk is characterized by a rich microbiota which constitutes a source for the infantile gut microbiota [90]. Except for initial observations of occurrence of microbes in milk in the 1950s, while studying the possibility of transmission of infections to infants via breastfeeding [91], human milk was thought of as a sterile fluid [92]. Around the beginning of the twenty-first century, a study in Spain by Martin and Colleagues [93] demonstrated, for the first time, the presence of the lactic acid bacterium, *Lactobacillus gasseri*, from milk and stools of mother–infant pairs, indicating that breast milk formed a potential source for infant microbiota. The non-sterility of breast milk has, ever since this initial observation, started to become extensively researched, and regarded as a protective factor arising from the wide range of microbes, in addition to the universally acknowledged nutritional role [94].

### 3.1. The Origin of Human Milk Microbiota

The origin of microbial populations in human milk is not completely understood and remains debatable. However, milk microbial populations have been proposed to originate endogenously, from the maternal digestive system through a complex pathway involving immune cells, or from the mother’s skin or infant’s mouth [95], or from the breast tissue itself [92]. It is plausible that more than one pathway contribute to the bacterial contents of milk [96].

#### 3.1.1. The Entero-Mammary Pathway

The discovery of anaerobic species, such as bifidobacteria, in human milk suggested the transfer of microbiota from the mother’s gut to milk, especially since these anaerobes are not commonly cultivated from breast skin swabs [90]. A physiological translocation mechanism is thought to carry maternal intestinal microbiota to the lactating mammary gland. This pathway is supported by the presence of bacterial communities in colostrum, collected even before first infant suckling occurs [97]. The unique process of translocation is aided by physiological and hormonal variations during late pregnancy and increased permeability of the intestinal epithelial lining [98], and is also immunologically mediated. Such active migration of microbes involves mononuclear cells, dendritic cells, and CD18 cells, delivering nonpathogenic bacteria from the intestine to the lactating mammary gland [99]. Dendritic cells are capable of penetrating the gut epithelium by loosening the tight junctions between intestinal epithelial cells and taking up bacteria from the gut lumen. Bacteria are then transported by macrophages to mesenteric lymph nodes and finally to the mammary gland [98,100].

#### 3.1.2. Retrograde Origin

Ultrasound imaging studies of nursing mothers have proved retrograde back flow of milk from the infant oral cavity due to infant suckling, suggesting a backward movement that transfers bacteria from infant’s mouth into the mother’s mammary gland [101]. This theory of milk microbiota origin is supported by similarities between the infant oral microbiota and human milk microbiota. For instance, *Streptococcus*, an abundant bacterial genus of breast milk, also dominates the salivary bacteria. Nevertheless, whether both bacterial communities share the same species and strains of Streptococcus remains to be determined [92,102].

#### 3.1.3. Transfer from Maternal Skin

This is a traditionally acceptable model of human milk microbiota origin, supporting that milk microbiota can result from contamination from maternal skin during the process of infant suckling, or a seeding process of milk from maternal skin. This theory is based on the similarities among the adult skin microbiota and milk microbiota, especially among the genera *Staphylococcus*, *Corynebacterium*, and *Propionibacterium* [103,104].

#### 3.1.4. Mammary Tissue Origin

In 2014, Urbaniak and Colleagues [105] showed, using culture and 16S rRNA gene sequencing, the presence of various genera of bacteria in breast tissue of Canadian and Irish women aged 18–90, with or without history of lactation, and without signs or symptoms of infection. Other investigations also showed that the breast tissue itself has an established microbiota, which is quite different from that of breast skin tissue and breast skin swabs [106]. Investigations on nipple aspirate fluid collected aseptically by application of negative pressure on the nipple, showed presence of bacterial DNA, probably from breast ducts [107]. It is probable that the existence of six to eight ductal openings on the surface of the human nipple permits microbes environmental microbes to access the ductal system of the breast [108]. It is also likely that such microbiota may influence the one present in human milk [92].

In summary, regardless of the relative contribution of the above four sources to microbes in milk, it has become generally acceptable that milk microbiota is not a secondary contamination, but rather a discrete microbiota, which is distinct from others in both the mother and the infant.

### 3.2. Types of Microbes in Human Milk

Both the early, culture-based methods, as well as the new, genomic-based and sophisticated techniques, such as next-generation sequencing, have been applied to analyze human milk microbiota and embrace its diversity [109]. Breast milk is a niche to several hundreds of bacterial species, and harbors about 1000 colony-forming units of bacteria/mL [110]. Following initial exposure to microbes upon birth, breast milk is the next immediate important source of various microbes to seed the infant’s gut [103]. As an evidence of this vertical transfer from milk microbiota to infant intestine, it was found that infants who primarily breastfeed during the first month of life, share 28% of their stool microbes with microbes of their mother’s milk. The number of shared microbes rises with the amount of daily breast milk intake in a dose-related manner, and microbes in the infant gut most resemble those from their own mother. The types and proportions of different microbes in human milk exhibit interindividual variation [103,111]. The major types are presented below.

#### 3.2.1. Bacteria in Human Milk

A very recent systematic review in 2019 determined the bacterial repertoire of human milk, showing the presence of about 820 species, mainly belonging to Gram-positive Firmicutes and Gram-negative Proteobacteria [112]. Firmicutes include members of the genera *Staphylococcus*, *Streptococcus*, *Lactobacillus*, *Bifidobacterium*, *Enterococcus*, *Veillonella*, *Gemella*, *Clostridium*, and others. On the other hand, Proteobacteria include *Escherichia*, *Pseudomonas*, *Enterobacter*, *Serratia*, *Ralstonia*, *Sphingomonas*, *Bradyrhizobium*, and others. Additionally, milk contains Actinobacteria, like *Actinomyces*, *Corynebacterium,* and *Propionibacterium*, Fusobacteria such as *Leptotrichia*, as well as Bacteroidetes, such as *Prevotella* [113]. Since 2011, scientific consensus has been molded towards the existence of a “core” bacteriome in human milk, consisting of the following nine genera: *Staphylococcus*, *Streptococcus*, *Serratia*, *Pseudomonas*, *Corynebacterium*, *Ralstonia*, *Propionibacterium*, *Sphingomonas*, and *Bradyrhizobium*. These represent about half of the microbial milk community, although their abundance might diverge among milk [87,102]. In terms of relative proportions, *Streptococcus* and *Staphylococcus* species are the most common, and this is universally true, irrespective of variability in geographic location or analytical methods of milk analysis, whether culture or molecular based. They were followed by *Bifidobacterium*, *Lactobacillus*, *Propionibacterium*, *Enterococcus*, and members of the Enterobacteriaceae family [114]. During breastfeeding course, the overall bacterial diversity decreases compared with colostrum and transition milk. Moreover, the composition of the bacteria shifts from skin and enteric organisms such as *Staphylococcus* and *Streptococcus*, to infant mouth and skin organisms, such as *Veillonella*, *Leptotrichia*, and *Prevotella*, which increase as lactation progresses and mature milk is secreted [115].

#### 3.2.2. Fungi in Human Milk

Most studies of human milk microbiome have overlooked fungi, and they have not been thoroughly assessed. In a cohort of Australian women, *Candida* was detected in breast milk in both culture and PCR-based methods [116]. Furthermore, a metagenomic analysis of milk samples showed not only fungal-related reads of DNA, but reads related to Archea and protozoa as well [117]. In 2020, a cohort of 271 samples of human milk revealed, through DNA sequencing, the presence of fungi in over 20% of samples, dominated by the genera *Candida*, *Alternaria*, and *Rhodotorula* [118]. In a study comparing transient and mature human milk samples, the most abundant fungal species in transient milk were *Saccharomyces cerevisiae* and *Aspergillus glaucus*. While *A. glaucus* was the second most common species in mature milk, *S. cerevisiae* disappeared, and *Penicillium rubens* appeared as the most abundant species, suggesting variable composition of milk mycobiome with maturity [119]. Further research is needed to establish the prevalence and role of fungi in human milk.

#### 3.2.3. Human Milk Virome

Recent evidence is emerging to show that the neonatal virome is modulated by breastfeeding [120], and human milk contains viruses transmitted from the mother to the infant to colonize the infant gastrointestinal tract [121]. These viruses include eukaryotic viruses, bacteriophages, and other viral particles [122]. Specifically, bacteriophages form a majority, and they have the ability to kill bacteria or provide them with potentially beneficial genes [109].

### 3.3. Milk Microbiota Diversity and Associated Factors

A myriad of environmental, genetic, and immune factors personalizes a mother’s milk microbiome. Among different women, and in the same woman experiencing diverse physiological, hormonal, and pathological conditions, an inconsistency is expected among communities of milk microbiota [92].

Primarily, maternal factors contribute to differences in milk microbiota composition. Khodayar-Pardo and Colleagues [123] showed that Cesarean section births were associated with total bacterial concentrations in milk higher than vaginal delivery, and significantly higher levels of *Streptococcus*, but significantly lower levels of *Bifidobacterium*. This was associated in some studies with risk of immune-mediated and inflammatory disorders in infants born by Cesarean delivery [124]. Possibly, physiological stress or hormonal signals of delivery could influence the microbial transmission process to milk [115]. The difference in composition of milk microbiota among vaginal deliveries and Cesarean section, however, was not significant in other investigations [125], warranting further analysis. A fail-safe mechanism has been proposed, whereby the mother passes along her bacterial imprint irrespective of how the baby is born, eliminating major differences in milk microbiome composition with mode of delivery [125]. The differences in milk microbiota among cesarean and vaginal deliveries are, however, thought to be only short lived when reflected on infant gut microbiome. By one month, vaginal and cesarean section infants cannot be separated on the basis of composition of their microbiota [126]. Based on available evidence, it is difficult to draw clear-cut conclusions regarding milk microbiota composition and both mode of delivery and milk maturity.

Maternal body weight also influences milk microbiota. Milk from obese mothers was found to include a bacterial community of lower diversity compared with milk from normal-weight mothers, according to findings of a randomized controlled trial, but was associated with higher numbers of *Staphylococcus* and lower numbers of *Bifidobacterium* [115]. The link between gut microbiota and obesity may possibly be extended to milk, substantiating the conception that obesity influences the milk microbiota via its influence on the gut microbiota [115,127]. Indeed, maternal diet influences milk microbiota, and this is elaborated in Section 3.4.

In 2015, and also relating to maternal factors on milk microbiota composition, Olivares and Colleagues [128] showed that levels of bifidobacteria were reduced in milk of mothers with celiac disease compared to healthy mothers, and this could, theoretically, diminish the protective effects of breastfeeding on the child’s risk of developing celiac disease. Further, breast milk favored *Clostridium leptum*, *Bifidobacterium longum,* and *Bifidobacterium breve* gut colonization, and these were associated with the HLA type of low genetic risk for celiac disease [129]. In an analysis of intrapartum antibiotic administration on human milk microbiota, it was found that the species *Bifidobacterium* was uniquely found in breast milk samples of mothers who did not receive antibiotics. Such observation may be significant, since reduced fecal bifidobacteria in early infancy may be associated with higher risk of non-communicable diseases in childhood, like atopy and overweight [130].

On another note, maternal postnatal psychosocial distress may alter maternal gut microbiota, which, in turn, may affect bacteria present in milk. Browne and Colleagues [131] found no significant differences in the relative proportions of major bacterial genera between women with high and low levels of psychosocial distress. However, progressive and distinct decrease in Firmicutes, Proteobacteria, and Bacteroidetes at the phylum level, and increase in *Acinetobacter*, *Flavobacterium*, and *Lactobacillus* at the genus level, were evident in milk samples of women with low psychosocial distress. High maternal psychosocial distress was also related to significantly lower bacterial diversity in milk three months after delivery. These findings suggest a likely relationship between maternal psychosocial distress and milk microbiota, indicating that post-partum psychological symptoms may impact infantile development and health, through their influence on milk.

Milk bacterial profiles do not appear to significantly differ in relation to maternal age, infant gender, or race/ethnicity in a given geographical region [109], but do differ across geographic locations of Europe, Africa, and Asia. Across these continents, Kumar and Colleagues [132] analyzed milk samples of 80 women from 4 different countries (China, Finland, South Africa, and Spain). Spanish women had highest abundance of Bacteroidetes, whereas Chinese women had highest abundance of Actinobacteria. Women who had a cesarean section had higher amount of Proteobacteria as observed in the milk of the Spanish and South African women. Interestingly, and in the emerging field of human milk mycobiome, a core of four genera was shared across milk samples from the above four countries, consisting of *Malassezia, Davidiella, Sistotrema*, and *Penicillium*, which are also present in the infant gut, supporting potential role of breast milk in the initial seeding of fungal species in the infant gut [133]. In a very recent investigation from Dubai [134], the genus *Hydrogenophaga* (of the beta-Proteobacteria), previously reported in breast cancer tissue [106], was significantly higher in the breast milk of local women compared to expatriates living in Dubai. This finding may shed a light on possible influence of race and lifestyle on human milk microbiota, but the importance of such data needs to be further explored.

Other determining factors of human milk microbiota have been investigated. In one survey, of milk samples collected at different stages of lactation, *Bifidobacterium* spp. concentration was significantly higher in milk samples from term gestations compared to preterm ones, indicating that gestational age plays a role in structuring milk microbiota [123]. This was not reproducible in another study on Canadian women, whereby comparison of bacterial profiles between preterm and term births showed no statistically significant differences [125]. The number of lactobacilli- or bifidobacteria-positive samples was significantly lower in women treated with antibiotics during pregnancy or lactation [135]. This emphasizes that consideration needs to be given to the impact of drugs administered to the breastfeeding mother, not only on potential consequences for the infant health, but also in diverting the normal milk microbiota [109].

To summarize the above, a review about the multitude of factors influencing human milk composition was very nicely and recently elaborated by Zimmermann and Curtis [136] in 2020. This review of 44 studies recognized some evidence that gestational age, infant sex, delivery mode, parity, lactation stage, diet, body mass index, composition of breast milk, geographic location, HIV infection, and method of collection affect composition of the breast milk microbiota. However, many studies were small and findings possibly conflicting, indicating need for further research setting some benchmarks on the internal and external players affecting the nature of human milk microbiota.

### 3.4. The Effect of Maternal Diet on Human Milk Microbiota and on Intestinal Microbiota of Infants

In addition to the numerous physiological, medical and environmental factors described above, it was shown that the assembly of microbes in human milk is also affected by maternal diet [137]. The associations between numerous macro- and micronutrients in maternal diet and the milk microbiota have been investigated.

In fact, maternal diet affects the concentration of certain substances, like fatty acids, in milk. In other words, maternal nutrient intake may indirectly help shape the bacterial community membership in human milk simply because of its impact on milk nutrient composition. For example, Kumar and Colleagues [132] documented multiple associations between human milk fatty acid profiles and variations in milk microbiota. Monounsaturated fatty acids of milk were negatively associated with Proteobacteria, but positively associated with *Lactobacillus* genus.

Additionally, maternal nutrient intake affects gastrointestinal bacterial communities, which in turn, may become part of the milk microbiota via the entero-mammary pathway (Section 3.1.1). For example, Carrothers and Colleagues [138] provided initial evidence for associations between maternal nutrition and maternal fecal microbial community structure during lactation. Increased intake of pantothenic acid, riboflavin, vitamin B6, and vitamin B12 were related to increased relative abundance of *Prevotella* and decreased relative abundance of *Bacteroides* in the maternal gut. Intake of copper, magnesium, manganese, and molybdenum were positively associated with Firmicutes and negatively associated with Bacteroidetes. Overall, findings steadily suggest that high consumption of a more nutrient-rich and calorie-loaded diet was positively associated with relative abundance of Firmicutes. It was suggested that such maternal dietary factors that directly influence the maternal gastrointestinal bacterial community, might also indirectly affect the milk microbiota [137].

Correlations between maternal dietary intake and milk microbiota composition have been validated by several studies. In one investigation from Brazil, postpartum women went through a validated food frequency questionnaire that covered the whole pregnancy period, and correlations were obtained between their diet and their milk microbiota. A global, significant association with milk microbiota diversity was observed for the intake of ascorbic acid during pregnancy [139]. In light of ascorbic acid importance, higher maternal consumption of citrus fruits, as well as of vegetables and β-carotene during pregnancy were protective against eczema in the offspring, in line with the positive effects of ascorbic acid on the human immune system [140]. Furthermore, positive correlations were found between *Bifidobacterium* in the milk and intake of polyunsaturated and linoleic fatty acids during lactation [139]. This is consistent with the conversion of polyunsaturated fatty acids to conjugated linoleic acid and conjugated linolenic acid, known to favor *Bifidobacterium* growth [141].

In a study of 21 healthy breastfeeding women at Washington State University and University of Idaho, diet records, collected over 9 dietary assessments over six months postpartum, were correlated with milk microbiota. The results showed that relative abundances of several bacterial groups were associated with changes in maternal dietary intake. For instance, intake of saturated fatty acids and monounsaturated fatty acids were inversely associated with the relative abundance of *Corynebacterium*; total carbohydrates, disaccharides, and lactose were negatively associated with Firmicutes; and protein consumption was positively correlated with the increase in *Gemella*, a genus belonging to the Streptococcaceae family [137].

In an interesting animal model of diet followed for 31 months, it was demonstrated that alone, diet may modulate mammary gland microbiota. Mediterranean diet resulted in approximately 10-fold increase in mammary gland *Lactobacillus* abundance compared with mammary tissue from Western diet-fed animals [142]. *Lactobacillus* is often thought of as commensal organism and is commonly included in probiotic formulations, where it directly contributes to beneficial nutritional, physiological, microbiological, and immunological effects in the host [143]. Again, with *Lactobacillus* transferred from maternal gut to human milk, and then from milk to intestinal tract of infants, a dynamic pathway exists in which maternal diet will play a major role in determining the profile of infant gut microbiota [144].

Another recent study reinforcing the hypothesis that maternal diet affects milk microbiota found, at one-month postpartum, significant negative correlation between *Streptococcus* in maternal milk and the intake of unsaturated fatty acids and the abundance of oleic acid. This was explained by the fact that streptococci may be sensitive to the antibacterial effects of fatty acids, causing a direct inhibition [145]. The same group reported that *Bifidobacterium*, pivotal in development of the infant gut microbiome, was detected, at extremely low abundances, in milk samples [145]. This observation is in favor of the current view that maternal milk provides a microbial source for colonization of the infant intestine. However, the main effect of milk on the baby’s gut composition does not necessarily come from the milk bacteria only, but rather from other milk components, as well, that help to enrich specific bacterial groups. This could explain why bacterial breast milk composition and fecal microbiota composition are not exactly the same, but correlations do exist [146]. In light of benefits of *Bifidobacterium*, a trial investigated maternal supplementation of this bacterium as well as *Lactobacillus* to maternal diet four weeks prior to the due date and for three months after delivery. The intervention did not significantly affect overall composition of breast milk microbiota transferred to the infant during breastfeeding, analyzed by 16S rRNA sequencing, thus questioning the substantial effect of probiotic supplementation to maternal diet on microbiota composition of breast milk [147].

The currently available reports on maternal diet and microbiota of human milk are still few and reports on the role of nutrients in the metabolism of bacteria in human milk are much needed. Furthermore, the somehow incomplete knowledge regarding the availability of macro- and micronutrients in milk, which are directly useful for bacteria, further complicate data interpretation. The relationship between maternal diet and milk microbiota has much yet to be investigated, and in-depth studies of various nutritive components and their concentrations on microbiota of milk are warranted.

### 3.5. Beneficial Effects of Milk Microbiota on Infant Health

The commensal and beneficial microbes in human milk play a pioneer role in shaping infant health, and perhaps represent an example of how intimate contact with the microbial world is necessary for normal development in early life [148]. Human milk microbiota affects the establishment of the largest human microbial reservoir in the gastrointestinal tract; also, the reciprocal relation between milk microbiota and gut microbiota is reflected on the development of immunity and protection against pathogens.

After birth, milk microbiota forms the most important determinant of infant gut colonization, which occurs in a stepwise fashion. With initial exposure to microbes transferred to the infant gut from mother’s milk, colonization of the intestine of infants starts, and may be essential for the maturation of the gut-associated lymphoid tissue, homeostasis of the intestinal epithelium, and development of intestinal physiology [149]. In the first few days after birth, the infant intestine is characterized by a heterogeneous population of microbes characterized by facultative anaerobes that belong to Enterobacteriaceae, *Streptococcus*, *Enterococcus*, and *Staphylococcus* that thrive on oxygen availability in the newborn gut. *Escherichia coli*, *Enterococcus faecalis*, and *Enterococcus faecium* are the most characterized species among first colonizers. Gradual oxygen consumption by such facultative anaerobes creates a reduced oxygen environment that allows expansion of obligate anaerobes such as *Bifidobacterium*, *Bacteroides*, and *Clostridium*. Intestinal colonization undergoes further changes upon introduction of solid food. The genus *Bifidobacterium* is detected in the first few months, predominates by 12 months, then declines towards the second year, at the end of which the infant microbiota becomes more diverse [150]. It is essential here to note the distinction between gut microbiota of breastfed and formula-fed infants: It is well known today that *Bifidobacterium* is the predominant intestinal genus in both feeding modes [151], with species variations. The species *Bifidobacterium longum*, *Bifidobacterium infantis*, *Bifidobacterium breve*, and *Bifidobacterium bifidum* are commonly detected in breastfed babies, while *Bifidobacterium adolescentis* and *Bifidobacterium pseudocatenulatum*, commonly seen among the intestinal adult microbiota, predominate in formula-fed babies [152]. Collectively, formula-fed infants in general have relatively stable and diverse microbial intestinal communities with higher levels of facultative anaerobes and strict anaerobes when compared to breast-fed infants. Fecal samples from breast-fed infants are less complex, with higher numbers of aerobic organisms, and with more critical changes in microbial composition up to the first year. Once weaning, or the introduction of solid foods into the feeding pattern, begins, differences in microbial communities between breastfed and formula-fed infants diminish, and the microbial profile shifts towards the adult intestinal microbiome [153].

The establishment and development of bifidobacterial and lactic acid bacteria in infant’s gut from viable inocula in human milk was demonstrated by several studies. For example, a study of Spanish full-term breastfed infants evaluated similarity between fecal bacteria and those from corresponding milk samples. It was shown that in one-day-old newborns, *Enterococcus* and *Streptococcus* were the microorganisms most frequently isolated, while from 10 days until 3 months of age, bifidobacteria became predominant. In corresponding breast-milk, *Streptococcus* genus was most frequently isolated, and *Lactobacillus* and *Bifidobacterium* were also obtained [154]. With an approximate viable bacterial density of 2–4 log colony-forming units/mL of human milk, resulting in a projected daily ingestion of 5–7 log cells with regular breastfeeding, it is expected that neonatal gut microbiota echoes the bacterial composition of breast milk [155]. A trend consistent with breastfeeding is that breast milk selects for a highly adapted intestinal microbiota, dominated by bifidobacteria and termed a “milk-oriented microbiota” [150]. When a disbalance occurs in such orientation health changes may result, for example, preterm infants with altered microbiota are susceptible to necrotizing enterocolitis due to the immaturity of their gastrointestinal and immune systems [48]. In 2020, a meta-analysis of necrotizing enterocolitis in premature infants showed that a mixture of *Bifidobacterium* and *Lactobacillus* could reduce morbidity, illustrating role of human milk microbiota in health and disease [156].

The human milk microbiota has a putative role in prevention of infections in newborns, and this occurs through its contribution to the competitive exclusion of pathogens and its involvement in the maturation of the immune system [155]. One possible example of the competitive exclusion of pathogens is the genus *Staphylococcus*. Several *Staphylococcus* species, especially *S. epidermidis*, colonize human milk and the intestine of breastfed infants. In an analysis of milk and stool samples from mother–infant pairs, Jiménez and Colleagues [157] found that *S. epidermidis* was the predominant species in the milk and stool of breast-fed infants, while it was less prevalent in those of formula fed-infants. The presence of adhesion-related genes in *S. epidermidis* was very high, while the biofilm-related operon *icaD* and the gene *mecA* were only detected only rarely the *S. epidermidis* strains. Hence, the bacterial attachment factors were present but less commonly the virulence or antibiotic resistance determinants. The commensal staphylococcal strains provided by breast milk to the infant gut may successfully compete with potentially harmful strains. In another, more recent investigation on intestinal *Enterococcus* from breastfed infants, the commensal enterococci harbored antibiotic resistance and virulence genes. However, the patterns of these genes were not consistent with those described for antibiotic-resistant hospital-associated enterococci, and none were resistant to vancomycin. The frequency of virulence determinants like hemolysin and gelatinase was also low, while some genes linked to colonization were abundant. Taken together, these findings suggest a possible benefit of enterococci in preparing the infant gut for effective opposition against pathogens [158]. It is possible that the pre-colonization of infants with maternal commensal strains will help later in preventing acquisition of infection by more virulent ones [155]. Interestingly, and in the field of HIV infection, lactobacilli cultured from human milk were able to inhibit HIV-1 infection in vitro by blockade of CCR5 co-receptor, and to a lesser extent CXCR4 or both coreceptors, suggesting a probable role for lactobacilli in mucosal protection against HIV-1 [159].

**Figure 2:**
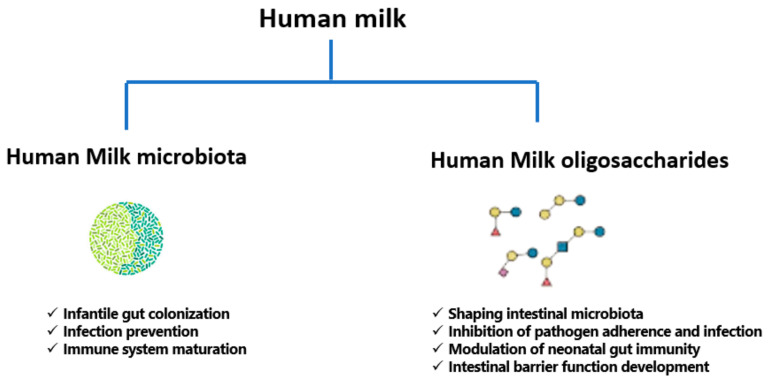
Functions of human milk microbiota and human milk oligosaccharides (HMOs).

In summary, an association between human milk microbiota, and consequently gut microbiota, and infant health and disease is an important contributor to infant hemostasis. In addition to the relationships described above, ongoing studies are continuing to investigate influence of microbiota on infant irritable bowel syndrome, inflammatory bowel disorder, and type 1 diabetes mellitus [160]. The functions of human milk microbiota and HMOs are summarized in Figure 2 below.

### 3.6. Probiotics of Human Milk

The term “Probiotic” means “for life” and it is currently used to name bacteria associated with beneficial effects for humans and animals. In 2001, the Food and Agriculture Organization of the United Nations (FAO) and the WHO defined probiotics as “live microorganism which, when administered in adequate amounts confer a health benefit on the host” [161]. This definition was revised in 2014 by the International Scientific Association for Probiotics and Prebiotics, including for probiotics “microorganism for which there are scientific evidence of safety and efficacy [162]. Probiotics that have been principally studied in humans include species from *Lactobacillus* and *Bifidobacterium* genera, both of which have been used safely for quite a long time [90]. Probiotic administration early in life may be effective in prevention and treatment of some disorders, leading to a correct microbial colonization while the gut microbiota is still being established [163]. Increasingly administered to infants, probiotics are intended to decrease the risk of certain ailments like necrotizing enterocolitis and late-onset sepsis in preterm infants, colic and antibiotic-associated diarrhea in term infants, in addition to some chronic diseases of childhood such as asthma and atopic disease [164]. The mechanisms responsible for probiotic action still require full elucidation; however, they include modification of the gut microbiota and normalizing its perturbation, competitive adherence to the mucosa and epithelium for pathogen exclusion, strengthening of the gut epithelial barrier, improving the digestion process by complementing the functions of absent digestive enzymes, and modulating the immune system to offer an advantage to the host [162,165]. There are agreed-upon criteria that a probiotic must harbor to be considered efficacious, such as capacity to survive in the GI tract, resistance to stomach acidity, lack of mobile antibiotic resistance genes, and demonstration of health benefits through efficacy testing followed by clinical trials [90,166].

Human milk-derived bacterial strains can be considered as potential probiotics; isolation of strains from milk for subsequent use in infant health and nutrition markets is used [90]. For example, human milk strains of *Lactobacillus reuteri*, a well-studied probiotic found in the gut and breast milk, can reduce the production of pro-inflammatory cytokines, promote T cell development, and decrease the microbial translocation across the intestinal epithelium from the gut lumen to tissues, thereby reducing inflammation. Notably, the decrease in *L. reuteri* in humans in the past decades was correlated with an increase in prevalence of inflammatory disorders. Direct supplementation of *L. reuteri* may be an attractive preventive and/or therapeutic model against inflammatory diseases [167]. In a murine model of asthma developed in 2020, Li and Colleagues [168] showed that oral administration of *L. reuteri* was more effective in asthma prevention than five other *Lactobacillus* species, where it reduced the risk of asthma by modulating specific gut microbiota to improve the immune environment of the lungs. *L. reuteri* supplementation, may, therefore, be a candidate against asthma and other allergic diseases. The same organism from human milk attenuated weight gain, fat accumulation, hypertriglyceridemia, and hypercholesterolemia in mice, thus opening a new horizon for the development of relevant foods to prevent metabolic disorders [169]. In a randomized controlled trial, *L. reuteri* administration was preventive in reducing pediatric consultations, parental discomfort, and the use of pain relievers for infant colic [170].

Apart from *L. reuteri*, *Lactobacillus fermentum* strains from human milk have been studied as probiotic candidates, and proved to be safe, well tolerated and useful for the prophylaxis against community-acquired infections [171]. They also showed cholesterol-lowering effects in simulated models of liver and gastrointestinal tract [172]. Both *L. fermentum*, and another milk-derived species, *Lactobacillus salivarius*, enhanced both natural and acquired immune responses, via activation of natural killer and T cell subsets and induction of a broad array of cytokines in peripheral blood mononuclear cells in vitro [173]. An interesting investigation of milk-derived *Lactobacillus casei* and *Lactobacillus paracasei* strains, demonstrated anticancer and antioxidant effect in HeLa cell lines of cervical cancer [174]. Likewise, anticancer potential was demonstrated by Rajoka and Colleagues [175] for *Lactobacillus rhamnosus* isolated from human milk, through induction of apoptosis and down-regulation of the *bcl-2* protooncogene, suggesting potential anticancer capability. A review of available studies on the use of probiotics in infantile acute gastroenteritis and antibiotic-associated diarrhea found that *L. rhamnosus* had the highest compelling evidence of efficacy in reducing duration of gastroenteritis by one day, and was the most effective probiotic in prevention of diarrhea [176].

As for bifidobacteria of human milk, studies are also available to support their use as probiotics. Maldonado and Colleagues [177] conducted a randomized controlled trial on the addition of a strain of *Bifidobacterium breve*, originally isolated from human milk, to infant formula. They concluded that this probiotic reduced crying rates, is safe, and induces beneficial effects on health. *Bifidobacterium longum*, isolated from human breast milk, had anti-oncogenic and tumor suppressor potential in murine colorectal cancer [178]. Park and Colleagues [179] showed that a probiotics formula containing both *B. longum* and *Lactobacillus acidophilus* alleviated fever, vomiting, and diarrhea with no adverse events in hospitalized infants with rotavirus infection.

Although *Bifidobacterium* and *Lactobacillus* strains from human milk persist as the most commonly sought bacteria for probiotic use, the health endorsing effects of other bacteria from milk is also under investigation. *Enterococcus faecium*, a probable probiotic isolated from breast milk was evaluated both in vitro and in vivo for its safety. The strain was non-hemolytic, sensitive to majority of antibiotics, and showed no alteration of normal growth and development in male rats. No adverse effects on general status nor behavior; additionally, no significant changes were noted in the hematological results, blood biochemistry, organ weights, and histopathology, and none of the vital organs of treated animals displayed signs of bacteremia nor infectivity. These findings indicated the candidature of *E. faecium* as a potential safe probiotic [180]. In another analysis of *E. faecium* and *E. faecalis strains* isolated from breast milk, these bacteria inhibited the growth of *Escherichia coli*, *Listeria monocytogenes*, *Salmonella typhi*, *Staphylococcus aureus*, *Shigella dysenteriae,* and *Streptococcus agalactiae*. However, phenotypic and genotypic virulence analysis indicated hyaluronidase enzyme production and vancomycin resistance in *E. faecalis*, calling for careful monitoring of probiotic strains for safety parameters [181]. Moreover, a study of cells and cytoplasmic fractions of *E. faecalis* and *Staphylococcus hominis* isolated from breast milk against MCF-7 breast cancer cell line revealed significant decrease in cellular proliferation in concentration- and time-dependent style. Morphological signals of apoptosis such as cell shrinkage, cell death, and membrane blebbing were observed in over one-third of the tumor cells [182].

In short, the available data on possible probiotics isolated from human milk is abundant. Further, focused evaluation of safety and efficacy of this rich milieu of microbes is needed to determine which members are good alternative nutraceuticals with health-promoting profiles or promising therapeutic indices.

## 4. A Review of Human Milk Oligosaccharides (HMOs)

In addition to the ideal balance of nutrients and the abundance of microbes present in human milk, rendering it a perfect food and an optimum medium for growth and immunity, multiple bioactive components also exist in this biofluid, such as immunoglobulins, cytokines, microRNAs, hormones, lactoferrin, and others [183]. Among these, perhaps HMOs constitute a major fraction and a supreme biochemical component that has prompted chemical, microbiological, and medical interest since the beginning of the twentieth century [184]. The major properties of HMOs and their benefits are discussed below.

### 4.1. Overview and Chemical Composition of HMOs

HMOs form a category of unconjugated, multifunctional, nondigestible, structurally diverse glycans that are unique to humans [185]. Quantitatively, HMOs represent an approximate 20% of total carbohydrate content of breast milk, and are the third largest solid component, after lactose and fats, amounting to 20–25 g/L in colostrum, and gradually decreasing to 5–15 g/L in mature milk [90,186]. The milk of mothers giving birth to premature newborns generally has higher HMO concentrations than that of mothers of term babies [187], although the concentrations were not significantly different in at least one report [188].

**Figure 3:**
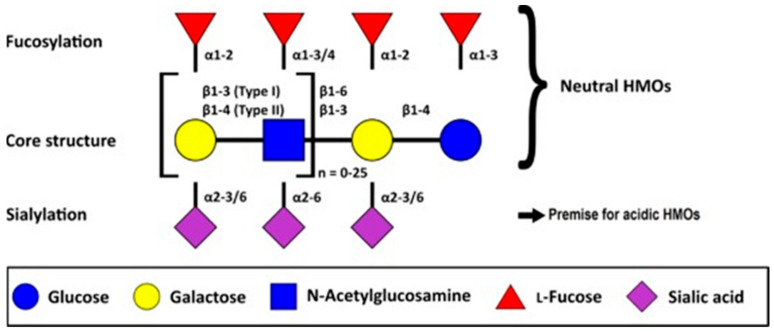
Basic structures of Human Milk Oligosaccharides (HMOs). The core HMO structures are shown in yellow and blue in the center of the figure. The upper panel shows fucosylated HMOs, and the lower panel shows sialylated ones. The building blocks are shown in the lowermost part of the figure [191].

Nowadays, over 200 HMOs have been identified, with variable structure and composition in milk between mothers and across the lactation period [183]. The chemical composition of HMOs is shown in Figure 3. Structurally, five monosaccharides form the building blocks of HMOs; these are D-glucose (Glc), D-galactose (Gal), N-acetylglucosamine (GlcNAc), L-fucose (Fuc), and sialic acid, which is exclusively found in humans as N-acetylneuraminic acid (Neu5Ac) [150,184,189]. All these monosaccharides are added to the precursor molecule lactose, which forms the core molecule, and are organized into various structures. The lactose core may be elongated enzymatically by β1-3 linkage to lacto-N-biose, or by β1-6 linkage to N-acetyllactosamine. The HMO structure can be elongated further via addition of: (1) lacto-N-biose and N-acetyllactosamine units (by β1-3 and β1-6 linkages); (2) Fuc connected with α1-2, α1-3, or α1-4 linkages; (3) and/or sialic acid residues attached by α2-3 or α2-6 linkages at terminal positions [190].

HMOs can be linear or branched. Elongation with lacto-N-biose appears to terminate the HMO chain, whereas N-acetyllactosamine can be further elongated by the addition of any of the two disaccharides. A β1-6 linkage between two disaccharide units introduces chain branching to HMOs. Branched structures are called iso-HMOs, while linear structures without branches are called para-HMOs. Lactose or the elongated oligosaccharide chain can be fucosylated with α1-2, α1-3, or α1-4 linkages, and/or sialylated with α2-3 or α2-6 linkages. Elongation or branching could lead to HMOs up to 15 monosaccharides long [192]. Nearly all HMOs contain lactose at the reducing end, which can be elongated by the addition of GlcNAc and Gal, forming either type 1 (Galβ1-3GlcNAc) or type 2 (Galβ1-4GlcNAc) chains in β1-3 or β1-6 linkages. Furthermore, Fuc and Neu5Ac can be attached to the HMO core or directly to the lactose reducing end [193]. HMOs can be subdivided into three fractions:

(a) A fraction of 35–50% of the total HMO content; these are neutral and contain fucose at the terminal position. These are called neutral (fucosylated) HMOs.
(b) A fraction of 42–55% of the total HMO content; these are neutral N-containing HMOs and contain N-acetylglucosamine at the terminal position. These are called neutral (nonfucosylated) HMOs.
(c) A fraction of 12–14% of total HMO content; these are acidic and contain sialic acid at the terminal position (e.g., 2′-sialyllactose). These are called acidic (sialylated) HMOs [192,194].

Fucosylated HMOs account for about 70% of total HMOs by weight, while sialylated HMOs account for 10–20% [195]. Examples of neutral and acidic HMOs and their basic structures are shown in Table 2 and Figure 3.

### 4.2. Milk Groups Related to Lewis Blood Group-Dependent HMOs: Definition and Relevance to HMOs Research

The HMOs composition and proportion are unique in the milk of each mother, being synthesized in the mammary gland by activity of specific glycosyltransferases, which sequentially add GlcNAc, Gal, Fuc, and Neu5Ac to the basic acceptor molecule, lactose [186]. The profile of HMOs from each mother reflects some blood group characteristics and appears to be genetically determined [192].

First, the link between HMO types and blood groups has been very well elucidated in literature, specifically pertaining to the Lewis blood group antigen [185]. By definition, blood group antigens are specific carbohydrate structures located on red blood cell surface, and constitute secondary gene products, where the primary gene products are the various glycosyltransferase enzymes that attach sugar molecules to the oligosaccharide chain [196]. Among various blood group antigens, the Lewis blood group system is encoded by the *Le* gene (also called the *FUT3* gene). In the mammary gland, the *Le* gene expresses a fucosyltransferase that adds Fuc in α1-4/3 linkage to synthesize fucosylated HMOs [197].

Second, another gene related to HMOs secretion is the secretor gene, designated by *Se*. Other blood group antigens, namely the A, B and H, are α1-2-linked fucose containing glycans present on red blood cells in individuals, representing respectively the blood groups A, B, and H. The enzyme fucosyltransferase 1 encoded by the *FUT1* gene is responsible for the synthesis of ABH antigens on red blood cells. However, the ABH antigens are also expressed in mucus, milk, and other secretions, where their expression is dependent on another enzyme, fucosyltransferase 2 (secretor type α1-2-fucosyltransferase) encoded by the *FUT2* gene [198]. Therefore, in the mammary gland, the *Se* and *Le* genes encode respectively the enzymes FUT2 and FUT3, which are involved in the biosynthesis of fucosylated HMOs [199].

Genetic mutations in Se and Le gene affect the synthesis of FUT2 and FUT3 enzymes, thereby affecting the chemical structure of HMOs. While secretor mothers with normal *Se* and *Le* genes secrete all types of HMOs, mutations in *Se* result in inactive FUT2; as such, milk from non-secretor (Se−) women contain no or very limited quantities of α1-2 fucosylated HMOs. Mutations in *Le* result in inactive FUT3; as such milk from Lewis-negative (Le−) women contains no or very limited quantities α1-4 fucosylated HMOs [200]. Depending on the activity of FUT2 and FUT 3 enzymes in the breast tissue, HMOs composition can be classified into four phenotypes, also referred to as four milk groups, as shown in Table 3 [193,201]. It is worth noting that the α1-3 fucosylated HMOs, such as 3′-fucosyllactose (3′-FL) and difucosyl-para-lacto-N-neohexaose (DFpLNnH), occur in all four SeLe groups, since their synthesis apparently is not affected by the *Se* and *Le* genes [199].

In a study comparing HMOs concentration across milk groups, the total amounts were highest in Se+Le+ milk at day 4 (23.4 g/L) and lowest in Se-Le- (11.3 g/L), with noticeable decline in concentrations in both groups during the first month of lactation (15% and 22%, respectively). Although decreased over time, the levels in Se+Le+ group continued to be highest within the first month, relative to all three groups, indicating that the concentration of total HMOs is linked to the maternal blood type characteristics [187]. While the prevalence of milk groups stated in Table above was detected by earlier studies, and mostly confirmed for European populations [201,202], a higher frequency of expression of recessive Lewis negative and non-secretors was detected in West African populations [194]. Also, a recent investigation from Dubai found the Se+Le+ type to be the most abundant (60%), followed by the Se+Le- type (23%). On the other hand, the non-secretor group was almost equally subdivided between Se–Le+ and Se–Le- groups [134].

In another recent study on breastfeeding Brazilian women [199], HMOs concentrations were highly variable even in women of the same SeLe phenotype, and unprecedented data on HMOs associations with maternal weight, infant’s weight, allergic disease, time postpartum, and sex were revealed. Allergic diseases in the mother, socioeconomic status, infant sex, and home pets were not associated with HMOs concentrations neither in Se+ nor in Se− women. In Se+ women, some positive correlations were observed between 2′-FL and maternal BMI. In the Se− group, significant correlations were mostly negative, involving 6′-SL and infants’ clinical variables such as gestational age, weight, and height.

Another hypothesis regarding normal HMO concentrations and profiles is that they might vary geographically. In a large observational study on health breastfeeding women from 11 nationalities, McGuire and Colleagues [195] reported that 3-fucosyllactose concentration was at least 4 times higher in milk collected in Sweden than in that collected in rural Gambia, while disialyllacto-*N*-tetraose (DSLNT) was about 4 times lower in Sweden compared to the latter. Maternal age, weight, and body mass index were correlated with several HMOs, and multiple differences in HMOs were shown between ethnically and genetically similar populations living in different locations, suggesting that the environment may play a role in regulating synthesis of HMOs. Targeted genomic analysis, as well as diligent examination of sociocultural, behavioral, and environmental factors is warranted to figure out the roles of these parameters in determining HMO profiles.

As such, both genetic and non-genetic factors influence HMOs composition and concentrations, and few studies have targeted this area. The HMO relations to Le and Se genotypes as well as other factors like gestational age, weight, parity, geographical location, gender, and lactation time remain challenging, especially with their effects on infant health, as described below.

### 4.3. Physiological Importance and Benefits of HMOs for Infant Health

With their unique and composite carbohydrate structure, HMOs resist gastrointestinal hydrolysis and digestion by gastric acidity and pancreatic and brush-border enzymes, hence are not absorbable in significant amounts. This allows HMOs to reach infant gut and produce a variety of beneficial effects [189].

#### 4.3.1. HMOs and Gut Microbiota Development

Essentially, HMOs serve as metabolic substrates for the growth of beneficial microorganisms in the infant intestine [90]. In infants who are breastfed, bifidobacteria predominate, and such dominance is the result of the presence of a bifidogenic agent in human milk. HMOs constitute such an agent, whereby they are fermented, in the large intestine, by bifidobacterial species [183]. The main products of this fermentation are acetic and lactic acids, which reduce the pH in the intestine, inhibiting the growth of pathogens. In addition, other short chain fatty acids are formed by fermentation, like butyric and propionic acid, where butyric acid is an important energy source for colonocytes. Hence, HMOs are the source of essential molecules needed for maintenance of intestinal health [203].

The ability of bifidobacteria to utilize HMOs depends on both intracellular and extracellular pathways [185]. *B. infantis* and *B. bifidum* deploy HMO utilization strategy, which involves translocation of intact oligosaccharides to the intracellular medium using transporters. Once internalized into the bacteria, HMOs are degraded via several intracellular glycosyl hydrolases, and the released monosaccharides are fermented producing ATP, and secreting acetate and lactate as end products. In contrast, the HMO consumption by B. bifidum depends upon initial extracellular processing, primarily via secreted glycosyl hydrolases that decompose HMO into mono- and disaccharides. The resultant products are then imported for intracellular catabolism [204]. When B. bifidum was incubated in HMOs-containing fecal cultures, the extracellular degradation mechanism produced other sugars that stimulated growth of different bifidobacteria. B. bifidum was able to mediate cross-feeding or sharing of HMOs degradants within bifidobacterial communities, increasing their diversity [205]. Recently, an analysis of different HMO degrading enzymes (glycosidase, phosphorylase, fucosidase, sialidase, galactosidase and others), transporters, and gene sets for HMO degradation was performed for at least six different species of bifidobacteria. The abundance of the HMO assimilation mechanisms and HMO-related genes was linked with *Bifidobacterium*-rich microbiota. Although the knowledge about HMO transporters is still incomplete, the accumulated data stress on the importance of varied HMO assimilation pathways for the formation of infant gut microbiota [206].

In addition to the influence of HMOs on bifidobacteria, other bacterial groups have also been studied. Thongaram and Colleagues [207] showed, using a high-throughput, low volume growth assay that L. acidophilus utilized extracellular β-galactosidase, encoded by lacL gene, to cleave the terminal galactose of LNnT for growth, leaving lacto-N-triose II in the medium. Schwab and Colleagues [208] investigated, in a human study, the interaction between bifidobacteria and Eubacterium hallii, one of the first butyrate producers in infantile gut. They demonstrated that E. hallii consumes acetate, lactate and 1,2-propanediol, which are fermentation products of HMOs by bifidobacteria, and eventually produces butyrate and propionate. These findings suggested E. hallii as a metabolically versatile species in infants, and which utilizes intermediates of bifidobacterial HMO fermentation. Taken together, these data provide insights into how HMOs shape the infant intestinal microbiota.

#### 4.3.2. HMOs and Infection Prevention

In addition to the nutritional role of HMOs on the intestinal microbiota of infants, there is increasing evidence that their interaction with pathogens is important in terms of reducing infection [186]. In doing so, and by being similar to human cell surface glycoconjugates utilized by microbes for attachment to intestinal cells, HMOs act as decoy receptors that bind pathogens [185,192]. For example, 2′-FL inhibits Campylobacter jejuni binding to intestinal mucosa both ex vivo and in vivo, and quenches the inflammation and sequalae induced by this bacterium, causing diarrhea to which young infants worldwide are most susceptible [209]. Furthermore, in a study about bioengineered 2′-FL and 3-FL, 2′-FL inhibited adhesion of *C. jejuni*, enteropathogenic *E. coli, Salmonella enterica,* and *Pseudomonas aeruginosa* to an intestinal human cell line. Moreover, adherence of *P. aeruginosa* to a human respiratory epithelial cell line was significantly prevented by 2′-FL and 3-FL [210].

Not only bacteria, but protozoa may be affected as well by the anti-adherence effect of HMOs in the infant intestine. HMOs were able to detach Entamoeba histolytica and rescue human intestinal epithelial cells from its destruction in a dose-dependent manner. The cytoprotective effects of HMOs were structure-specific, where Lacto-N-tetraose rescued up to 80 % of the cells, while HMOs with fucose α1-2-linked to the terminal galactose did not show an effect. Galacto-oligosaccharides (GOS), which are added to infant formula to mimic some of the HMOs beneficial effects, abolished *E. histolytica* attachment and cytotoxicity. These results explain why breast-fed infants have lower risk *of E. histolytica* infections, and suggest HMOs and GOS as well-tolerated, safe, stable, and valuable alternatives to anti-amoebic agents [211]. HMOs also inhibit infectivity of reoviruses, by blocking their attachment to host cells, and this blockade is serotype-specific [212].

More recently, it has been documented that some HMOs exhibit bacteriostatic properties against group B Streptococcus (GBS). GBS is known to be a leading cause of invasive bacterial infections in newborns, and is vertically acquired during childbirth, secondary to colonization in the vagina. Specific non-sialylated HMOs, in synergy with specific conventional antibiotics agents, may modify particular GBS components in a mode that impairs growth kinetics [213]. In growth and biofilm assays, HMOs showed antibacterial and antibiofilm activity, not only against GBS, but also against *Acinetobacter baumannii* and *Staphylococcus aureus* [214]. Additionally, in a quantitative assay of fungal invasiveness, HMOs were effective in prevention of the invasion and damage of the premature infant intestine by *Candida albicans* hyphae, perhaps providing a model of reducing fungal pathogenesis [215].

#### 4.3.3. HMOs and Immunomodulatory Effect

The immune system of the infant is functionally immature and naïve, and HMOs may play a role in neonatal gastrointestinal and systemic immune development and physiology [216]. The importance of HMOs may be explained, not only by inhibiting the adhesion of microorganisms to the intestinal mucosa, but also by the expression of genes that are involved in inflammation [192]. HMOs can bind to cell surface receptors expressed on epithelial and immune system cells, and thereby modulate neonatal immunity in the infant gut [217]. For example, in a study of HMO effect on colonic epithelial cells, HMOs influenced the expression of multiple cytokines and chemokines, adhesion molecules and receptors. These results suggested that HMOs have a valuable contribution to the development and maturation of the intestinal immune response [218].

To explore the effect of HMOs on the components of immune signaling, human milk was tested on enterocyte cell lines treated with pro-inflammatory molecules, namely, tumor necrosis factor-α (TNF-α) or interleukin-1β (IL-1β), or infected with *Listeria* or *Salmonella*. HMOs reduced TNF-α- and IL-1β-induced inflammatory responses to 25–26% and pathogen-induced IL-8 to 36–39%. HMOs reduced the inflammatory response to *Salmonella* infection by the intestinal tissue, suggesting them as strong physiologic anti-inflammatory agents in human milk, and as contributors to innate immune modulation [219].

In terms of effect on immune cells, HMOs isolated from pooled human milk, induced semi-maturation of human monocytes-derived dendritic cells, and elevated levels of IL-10, IL-27 and IL-6 but not IL-12p70 and TNF-α, thereby playing a modulatory role in development of the neonatal immune system [220]. Recently, 2′-FL, abundantly available in human milk, enhanced Th1-type IFNγ and regulatory IL-10 secretion of peripheral blood mononuclear cells. Dendritic cells exposed to 2′-FL instructed the secretion of IFNγ and IL-10 from CD4+ T-cells, suggesting the growth of a regulatory Th1 response [221].

The effect of HMOs on the immune system may be, as well, associated with the effect of particular receptors. Galectins are glycan-binding proteins involved in intracellular signaling, cell–cell communication processes, cellular proliferation and survival. As endogenous lectins, they are expressed and released by different cell types, including immune, tumor, and endothelial cells [222]. Some HMOs contain β1-3- or β1-4-linked galactose at the non-reducing end, which can be the potential target for galectin-mediated interactions. The binding affinities of 31 free HMOs with the galectins Gal-1, Gal3, and Gal-7 were studied by catch-and-release electrospray ionization mass spectrometry (CaR-ESI-MS). In such analysis, HMOs showed high binding affinity to galectins. Whether HMOs are capable of modulating galectin-mediated immune responses still needs further study but galectins may prove as potential HMO receptors for immune system mediation [223].

#### 4.3.4. HMOs and Intestinal Barrier Function

One important function of HMOs is to promote development of gut barrier function, where the gastrointestinal tract is capable of digestion and absorption of nutrients, while simultaneously acting as a barrier against intruders and toxic agents [185]. HMOs support the intestinal barrier function, initially immature at birth, indirectly by influencing composition of the microbiota, and directly through modulation of intestinal cells. HMOs, through metabolism into short chain fatty acids and cross feeding with butyrate-producing bacteria, induce butyrate production in the colon. Butyrate is an essential metabolite, as it forms the preferable energy source for colon epithelial cells, contributes to the preservation of the gut barrier, and displays immunomodulatory effects [224]. Therefore, HMOs support barrier function through promotion of the formation of fermentation products, especially by *Bifidobacterium* [225].

Another possible pathway of intestinal barrier evolution by HMOs is stimulating the development of the epithelial glycocalyx on gut epithelial cells, which can contribute to gut homeostasis and form a physical barrier on top of the gut epithelium. The glycocalyx present on the neonatal gut epithelium offers binding sites for commensals and may prevent adhesion of pathogens as well as serve as a barrier for toxins and enzymes. If the glycocalyx components are not properly developed, this may elevate the risk of gastrointestinal disorders [226]. In 2019, Wu and Colleagues [227] demonstrated that HMOs increase mucin levels and decrease bacterial attachment in a murine model of necrotizing enterocolitis. HMOs directly induced the expression of chaperone proteins including protein disulfide isomerase, whose suppression removed the protective effects of HMOs on barrier function as well as on necrotizing enterocolitis protection in vivo. Such results provided insights to the possible mechanism by which HMOs protect the neonatal intestinal barrier through upregulation of mucins. The aforementioned evidence suggests that HMO supplementation may be a valuable approach to build up the gut barrier and perhaps restore or strengthen its function in conditions associated with occurrence of a ”leaky gut” in humans. Such condition includes ageing, irritable bowel syndrome, or inflammatory bowel disease. However, the exact mechanism of these effects remains to be largely explored [224].

### 4.4. HMOs and Prebiotics

Introduced in 1995, the concept of a prebiotic was described as “a non-digestible food ingredient that beneficially affects the host by selectively stimulating the growth and/or activity of one or a limited number of bacteria in the colon, and thus improves host health” [228]. This definition, armed with major scientific and clinical developments, has significantly evolved over the past two decades [229], until, it was expanded in 2017 by The International Scientific Association for Probiotics and Prebiotics (ISAPP), which currently uses an updated definition for a prebiotic as “a substrate that is selectively utilized by host microorganisms conferring a health benefit” [230].

Prebiotics must be resistant to gastric acidity, to hydrolysis by host enzymes and to gastrointestinal absorption. HMOs have the interesting feature of meeting all three criteria [183]. In fact, research has shown that HMOs, specifically 2′-FL, are even more powerful than standard commercial prebiotics, such as fructo-oligosaccharides and have multiple functions, including immune, gut, and cognition benefits [231]. Despite their beneficial effects, among HMOs, only 2′-FL, LNnT, and 3′-SL are currently added to infant formula, mainly because the complexity of HMOs makes their commercial synthesis still challenging and expensive (Table 4). 2′-FL was a logical starting point for HMO production due to its abundance in milk and its simple structure. LNnT is less abundant but is easier to synthesize. Nondigestible fibers such as GOS and inulin-type fructans are nowadays added to commercially available infant formulas to provide an alternative to HMOs and their benefits [185,232]. However, advances in HMO synthesis are desirable, and various disciplines have been utilized to prepare single HMO compounds by chemical or enzymatic syntheses or by whole-cell biotransformation using recombinant bacterial cells. These attempts utilize lactose as an acceptor molecule, and fucosyltransferases to produce 2′-fucosyllactose, 3-fucosyllactose, or more complex fucosylated core structures. Sialylated HMO can be produced by enzymatically using sialyltransferases and trans-sialidases. Core structures as lacto-N-tetraose can be obtained by glycosyltransferases from chemical donor compounds or by multi-enzyme cascades [191]. Whole cell biotransformation [233] and recombinant techniques [234] are also additional promising options to synthesize HMOs.

Although attempts to synthesize HMOs represent an initial phase in tapering the compositional gap between human milk and infant formula, it is unclear whether one or two HMO molecules will be capable of inducing the complex actions exerted by the rich blend of HMOs that breastfed infants ingest in human milk. Interestingly, 2′-FL, LNnT, and combinations of both used in a simulated ecosystem of adult human intestine were shown to increase bifidobacterial levels and to decrease levels of IL-6 and cellular permeability [224]. Still, a low diversity of HMOs in breast milk was associated with necrotizing enterocolitis in infants fed exclusively with breast milk [235]. Thus, as more HMOs become commercially available from chemical, microbial, or biotechnological synthesis sources, it is anticipated that more oligosaccharides will be added to infant formula, whether as single compounds or in synergy with additional prebiotics [216]. Plus, the use of human milk-based fortifiers having a full spectrum of HMOs, similar to that in breast milk, is quite tempting to nourish infants and support their gut development and maturity and warrants further investigation. Moreover, the capacity of HMOs to modulate the immune function and to reinforce the gut barrier might support their ability to afford health paybacks in adults, rather than in infants only [224]. Despite that the focus on HMO supplementation to date has been on infants, the transfer of HMOs to adults as well seems on the rise, and commercialization of HMOs is slowly, but surely, coming to make a case for HMO adult supplementation.

## 5. Milk Microbiota and HMOs: Are There Any Correlations?

As stated above, the composition of human milk is dependent on genetics, diet, lifestyle, lactation period, and other factors. Certain milk components, such as HMOs and bacteria seem also to vary depending on diet, delivery mode, geographical area, and other factors. However, the complex microbiota–HMOs interactions, and their consequences for infant’s health on both short- and long-term, stay uncertain [236]. To put it in a simple manner, milk metabolites, especially HMOs, may influence milk microbial communities, and these, in turn, influence intestinal microbiota of infants, probably favoring specific bacterial genera [237]. However, the interplay among these components is yet to be understood. In recent years, investigations aiming at characterization of relationships among HMOs and bacteria in milk has been ongoing.

In their study of the milk microbiota and HMO relationships in women from several areas of the northwest regions of the US, Williams and Colleagues [237], found that total HMOs and 2′-FL were positively correlated with relative proportion of Staphylococcus, while 3′-SL was inversely correlated with proportions of *Ralstonia* and *Novosphingobium*. However, with only 16 milk samples analyzed, the authors suggested that a more thorough interpretation of the repercussions of various HMOs on milk microbiota is needed. In a recent pilot observational study on Spanish breastfeeding women, the predominant HMOs in milk of secretor mothers were 2′-FL and lacto-N-fucopentaose I, whereas the milk of non-secretor mothers was characterized by lacto-N-fucopentaose II and lacto-N-difucohexaose II. Differences in microbiota types and concentrations were related to secretor/non-secretor status. Lactobacilli, enterococci, and streptococci were lower in non-secretor compared to secretor samples, while *Bifidobacterium* was less prevalent in non-secretor samples. Despite little differences on diversity and richness, non-secretor samples had less Actinobacteria and more Enterobacteriaceae, Lactobacillaceae, and Staphylococcaceae. This study supported the limited data on the complex interaction between microbiota and HMO during lactation, specifically with secretor status, although the potential biological influence on the newborn remains indiscernible [238]. In human colostrum of women from Finland, HMO content analyzed by high-pressure liquid chromatography and microbiota types analyzed by quantitative PCR, showed higher total HMO concentration associated with higher counts of *Bifidobacterium*. Furthermore, positive correlations were observed among sialylated HMOs and B. breve, and among non-fucosylated/non-sialylated HMOs and *B. longum*. Furthermore, positive correlations were observed among fucosylated HMOs and Akkermansia muciniphila, and among fucosylated/sialylated HMOs and *S. aureus*. Both oligosaccharides and microbes provided a concise inoculum that will provide for the compositional development of the infant gut microbiota [239]. In another study, lactobacilli and bifidobacteria, obtained from commercial probiotic supplements or from culture collections, were capable of fermentation of HMOs. *B. longum* ssp. *infantis* and *B. infantis* were able to ferment 3′-sialyllactose, 6′-sialyllactose, 2′-fucosyllactose, and 3′-fucosyllactose. B. infantis degraded almost 90% of 2′-fucosyllactose but left most of the fucose, as revealed by HPLC. Among bifidobacteria, only the *B. infantis* strains and *B. breve* were able to ferment lacto-N-neotetraose (LNnT). On the other hand, among lactobacilli, *L. acidophilus* was the most efficient at utilizing LNnT. The extracellular β-galactosidase of L. acidophilus, encoded by lacL, cleaved the terminal galactose of LNnT for growth, leaving lacto-N-triose II in the media. Moreover, the inactivation of lacL gene abolished growth of *L. acidophilus* on LNnT [207]. Not only do these results underwrite the increased scientific awareness of HMO-microbiota interactions, but they also emphasize the promise for prospective combinations of probiotics and HMOs, in a likely analogy to human milk constituents.

To ascertain the interaction between metabolomic profile of milk, including HMOs, and milk microbiota, an investigation on milk from different populations analyzed milk content using nuclear magnetic resonance spectroscopy (NMR). Specific interrelations between HMOs and milk microbiota were identified. Gammaproteobacteria positively correlated with lacto-N-fucopentaose I, and 2-fucosyllactose, although these HMOs negatively associated with Alpha and Betaproteobacteria and Bacilli. Further, the Actinobacteria in the human breast milk were negatively associated with lacto-N-fucopentaose I and 2-fucosyllactose acetate. These findings are in agreement with the existing, although limited data on complex relations between the milk microbiome and various types of HMOs during lactation [236]. In milk of mothers from Dubai, negative correlations were detected for the genus *Atopobium* versus sialyl-lacto-N-tetraose, for *Leptotrichia* and *Veillonella* versus 6′-sialyl-lactose, and for *Porphyromonas* versus lacto-N-hexaose. It could be hypothesized that, because they were able to use that particular HMO, they proliferated in the mammary gland; this, nevertheless, remains to be investigated [134]. In another investigation, *Streptococcus* and *Staphylococcus* showed an increase in relative abundance in relation to high consumption of the fucosylated HMOs, namely, DFL, LNFPII, and LNFPIII. The presence of HMOs may stimulate the growth of breast milk-associated *Staphylococcus* by activating growth-promoting signals without being actively metabolized by this strain. *Streptococcus* and *Staphylococcus* that cross-feed on HMO metabolites may play a role as well, although at the present time, no studies show direct evidence of that [240].

Scientific research on associations between milk microbiota profiles and HMOs has sometimes elucidated mechanisms of HMO utilization, rather than only the correlations. Perhaps the best example of this is the utilization of HMOs by *Bifidobacterium* in breast milk, which has been relatively well described. The dominant Bifidobacterium species can utilize various HMO components as their solitary carbohydrate source [241]. These species basically include strains of *B. bifidum, B. longum* subsp. *infantis*, and *B. breve*. *B. bifidum* extracellularly hydrolyzes complex HMO structures, including LNT and LNnT, using glycosyl hydrolases, followed by internalization and intracellular degradation and metabolism of the resulting mono- and disaccharides, such as LNB. Furthermore, *B. longum* subsp. *infantis* internalizes intact LNT, LNnT, and LNB, then uses a series of sequential hydrolytic/phosphorolytic reactions starting from the nonreducing end of the carbohydrate structures for degradation and metabolic processing. However, *B. infantis* is also known to take up and utilize fucosyl- and sialyl-lactose [242]. To this end, a multicomponent transcriptional regulation system that controls the recently identified pathways of HMO metabolism was described in B. breve. These include three transcriptional regulators, with the corresponding operator and associated promoter sequences, the latter governing the transcription of the genetic elements involved in LNnT/LNB metabolism. This result, not only provides some vision of the regulatory mechanisms present in bifidobacteria, but also offers an example of a system of consecutive steps regulating microbial carbohydrate metabolism [243].

The above results show high variability in HMOs and milk microbiota profiles, with possible existing correlations. It is anticipated that larger trials, more diverse populations, and targeted genomic analyses will elucidate such correlations in a more definitive manner. These trials should also be able to establish whether such correlations are dependent on genomic, sociocultural, or environmental features.

## 6. Future Implications in Milk Microbiota and HMOs Research

It is of no doubt that human milk harbors extraordinary features which make it a treasured fluid, whose amazing assets and roles are yet to be fully clarified. Research on human milk will continue to elucidate additional benefits and correlations among components, and such area of scientific investigation is very promising. Perhaps the driving force for such research lies in the incessant attempts to produce an industrial infant formula that mimics the nutritional breast milk composition as much as possible, to be able to substitute breastfeeding when this process is not possible or is solely inadequate [244]. Randomized controlled trials on milk benefits continue to be realized, and some effects under investigation are the analgesic effect of human milk [245] or milk odor [246,247], and the lessened need for antibiotics up to the first year of life [248].

In the field of milk microbiota and HMOs, characterization of the valuable organisms of the human microbiota and their utilization of defined HMOs on a strain-specific basis is essential. Furthermore, the number of randomized trials that have assessed the impact of HMOs on infant health is very limited. Furthermore, the number of subjects studied for determination of the safety and tolerance of HMO-supplemented formulas is not enough to generate actual evidence of the potential prophylactic role of HMOs against infection [192]. As previously mentioned, limited supply of HMOs currently impedes extensive trials on these compounds to develop targeted HMO-based prebiotic therapy [249,250]. Continued development of large-scale HMOs synthesis via different enzymatic engineering pathways should facilitate human trials, especially interventional ones, in the coming years [251]. New modalities of synthesis such as loop engineering [252] and the use of specific fucosidases [253,254] to synthesize specific HMO structures are being explored. Such new means, coupled with increased understanding of the dynamic interrelationship between HMO structure and the developing intestinal microbiota, shall enable the design and testing of novel prebiotic agents based on HMOs in clinical settings in the near future.

## 7. Conclusions

In brief, human milk encompasses a wealth of biologically active components that contribute to multiple advantages, far beyond infant nutrition. The past decade has witnessed an explosion of research and interest in the human milk microbiota. This fluid represents a unique resource for translational medicine, from research on the bench into clinical applications such as pro- and pre-biotics [255]. With its rich assortment of biologically active molecules and demonstrated benefits, both in newborn life and later into adulthood, ongoing characterization of the mechanisms through which milk components enhance development, shaping of microbiota, and immunity is on the rise. Additional investigations regarding links between the human milk microbiota and remarkably bioactive molecules, such as HMOs, are highly warranted.

## Figures and Tables

**Table 1:** Main nutrient constituents of human milk and respective concentrations [8,9,10,11].

**Macronutrients**	
Protein	0.9 g/dL
Fat	3.5 g/dL
Carbohydrates (mainly glucose)	6.7 g/dL
**Minerals**	
Calcium	200–250 mg/L
Magnesium	30–35 mg/L
Phosphorus	120–140 mg/L
Sodium	120–250 mg/L
Potassium	400–550 mg/L
Iron	0.3–0.9 mg/L
Fluoride	4–15 µg/L
**Fat-Soluble Vitamins**	
Vitamin A	0.3–0.6 mg/L
Vitamin D	15 IU/day (in exclusively breastfed infants) or 0.33 µg/L
Vitamin E	3–8 mg/L
Vitamin K	2–3 µg/L
**Water-Soluble Vitamins**	
Ascorbic acid	100 mg/L
Thiamine (vitamin B1)	200 µg/L
Riboflavin (vitamin B2)	0.35–0.39 mg/L
Niacin	1.8–6 mg/L
Vitamin B6	0.09–0.31 mg/L
Folate	80–140 µg/L
Vitamin B12	0.5–1 µg/L

**Table 2:** Examples of the three different fractions of HMOs, showing neutral (fucosylated and nonfucosylated) and acidic compounds.

Neutral Fucosylated HMOs	Neutral Nonfucosylated HMOs	Acidic HMOs
2′-Fucosyllactose (2′-FL)	Lacto-N-biose (LNB)	Disialyllacto-N-tetraose (DSLNT)
3-Fucosyllactose (3-FL)	Lacto-N-tetraose (LNT)	Sialyllacto-N-neo-tetraose b (LST b)
Lacto-N-fucopentaose I (LNFP I)	Lacto-N-neotetraose(LNnT)	Sialyllacto-N-neo-tetraose c (LST c)
Lacto-N-fucopentaose II (LNFP II)	Lacto-N-hexaose (LNH)	Sialyl lacto-N-tetraose a (LST a)
Lacto-N-fucopentaose III (LNFP III)	Lacto-N-neohexaose (LNnH)	3′-Sialyllactose (3′-SL)
Difucosyllactose (DFL)		6′-Sialyllactose (6′-SL)

**Table 3:** The four different milk groups based on genotypes of *Le* and *Se* genes, with prevalence, type of linkages in HMOs, and HMO examples. Prevalence is dependent on data from Thurl et al. and refers to the European population [201].

Milk Group	Prevalence	Fucosylated HMO Linkages	Examples of Fucosylated HMOs
Se+Le+	70%	α1-2 α1-4 α1-3	2′-Fucosyllactose (2′-FL) Lacto-N-difuco-hexaose I (LNDFH I) Lacto-N-fucopentaose II (LNFP II) 3′-Fucosyllactose (3′-FL) Difucosyllactose (DFL)
Se-Le+	20%	α1-4 α1-3	Lacto-N-difuco-hexaose II (LNDFH II) LNFP II 3′-FL
Se+Le-	10%	α1-2 α1-3	Lacto-N-fucopentaose I (LNFP I) 2′-FL 3′-FL
Se-Le-	1%	α1-3 only	Difucosyl-para-lacto-N-neohexaose (DFpLNnH) 3′-FL

**Table 4:** The three HMOs approved for use in infant formula, with dates of approval by the European Food Safety Authority and the US Food and Drug Administration.

HMO	Structure	Approval in EU (European Food Safety Authority)	Approval in USA (US Food and Drug Administration)
2′-Fucosyllactose (2′-FL)	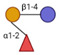	2015	2016
Lacto-N-neotetraose (LNnT)	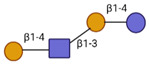	2015	2016
3′-Sialyllactose (3′-SL)	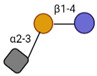	-	2019

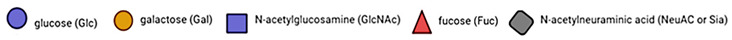
.
